# Relationship between adult height and body weight and risk of carotid atherosclerosis assessed in terms of carotid intima-media thickness: The Nagasaki Islands study

**DOI:** 10.1186/1880-6805-32-19

**Published:** 2013-11-01

**Authors:** Yuji Shimizu, Mio Nakazato, Takaharu Sekita, Koichiro Kadota, Kazuhiko Arima, Hironori Yamasaki, Hisashi Goto, Satoshi Shirahama, Noboru Takamura, Kiyoshi Aoyagi, Takahiro Maeda

**Affiliations:** 1 Department of Community Medicine, Nagasaki University Graduate School of Biomedical Science, Nagasaki, Japan; 2 Department of Island and Community Medicine, Nagasaki University Graduate School of Biomedical Science, Nagasaki, Japan; 3 Department of Public Health, Nagasaki University Graduate School of Biomedical Science, Nagasaki, Japan; 4 Center for Health and Community Medicine, Nagasaki University, Nagasaki, Japan; 5 Goto Health Care Office, Nagasaki, Japan; 6 Kamigoto Hospital, Nagasaki, Japan; 7 Department of Global Health, Medicine and Welfare, Nagasaki University Graduate School of Biomedical Sciences, Nagasaki, Japan

## Abstract

**Background:**

Previous studies have reported an inverse association between height and risk of cardiovascular disease. However, evidence is limited for the association between risk of atherosclerosis and height. Further, although the association between atherosclerosis and body mass index (BMI) is reportedly positive, there have been no reports of studies on associations between height and atherosclerosis in relation to BMI.

**Methods:**

We conducted a cross-sectional study of Japanese men aged 30 to 89 years undergoing general health check-ups.

**Results:**

Of the 1,337 men, 312 were diagnosed with carotid atherosclerosis (carotid intima-media thickness (CIMT) ≥ 1.1 mm), but no significant association was found between height and carotid atherosclerosis for the entire study group. Stratification by BMI status of those analytical findings disclosed a significant inverse association between height and carotid atherosclerosis among overweight (BMI ≥ 25 kg/m^2^) but not among non-overweight (BMI < 25 kg/m^2^) men. The classical cardiovascular risk factors-adjusted odds ratio (OR) and 95% confidence interval (CI) of carotid atherosclerosis for an increment of one SD (standard deviation) in height (6.70 cm) were 0.71 (0.54 to 0.94) for overweight (BMI ≥ 25 kg/m^2^) and 1.05 (0.87 to 1.27) for non-overweight (BMI < 25 kg/m^2^) men.

**Conclusion:**

Independent from classical cardiovascular risk factors, height was found to be inversely associated with carotid atherosclerosis for overweight but not for non-overweight men.

## Introduction

Several prospective studies have demonstrated that height is inversely associated with incidence of or mortality from cardiovascular disease [1,2].

Another study has reported that carotid intima-media thickness (CIMT), measured non-invasively with high-resolution ultrasound scanning, is a well-known indicator of generalized atherosclerosis and strongly associated with risk of cardiovascular disease [3]. However, there have been no reports of studies on the relationship between height and CIMT among Japanese people. The finding of a previous study that CIMT was positively associated with an increase in body mass index (BMI), even for the moderately overweight [4], suggests that the associations between height and atherosclerosis assessed in terms of CIMT, may be strongly affected by BMI status. Nevertheless, there have been no reports of studies on associations between height and carotid atherosclerosis assessed in terms of CIMT and in relation to BMI. On the assumption that height is an easily measured variable, and is thought to be determined during childhood and adolescence by genetic predisposition, nutrition, physical and social environments, as well as other factors, whereas body mass index (BMI) is regarded as a surrogate marker of current physical condition, and higher BMI is known to be a classical cardiovascular risk factor [5], we used cross-sectional data for community-dwelling Japanese men to investigate the association between height and carotid atherosclerosis in terms of BMI status.

We therefore investigated such associations among Japanese men who participated in a general medical check-up between 2005 and 2012.

## Methods

### Subjects

This study was approved by the Ethics Committee for Use of Human Subjects of Nagasaki University (project registration number: 0501120073).

The survey population included 1,538 men aged 30 to 89 years, all residents of the western rural community of the Goto Islands, who participated with their written agreement in this study between 2005 and 2012. A total of 46 individuals with missing data and 155 individuals with a history of cardiovascular disease were excluded, leaving 1,337 men for enrollment in this study (total participation rate was more than 90%).

The mean age of the study population was 64.8 years (±10.7 SD; range 30 to 89). Body weight and height were measured with an automatic body composition analyzer (BF-220; Tanita, Tokyo, Japan) at the time blood was drawn.

### Data collection and laboratory measurements

Fasting blood samples were obtained and the serum was separated and centrifuged after blood coagulation. Serum samples were also collected in a siliconized tube.

Serum triglycerides, serum HDL-cholesterol, serum aspartate aminotransferase (AST), HbA_1c_ and serum creatinine were measured with standard laboratory procedures. Since the method recently proposed by a working group of the Japanese Chronic Kidney Disease Initiative (JCKDI) [6] does not take the influence of height and weight into account, whereas Horio’s method [7] does, we estimated the glomerular filtration rate (GFR) by using both methods. The first method was established with three variables, resulting in:

$$\begin{array}{l} \mathrm{GFR}\;\left(\mathrm{JCKD}\right)\;\left(\mathrm{mL}/\min/1.73\;m^{2}\right)\\ =194\times {\left(\text{Serum}\;\text{creatinine}\;\left(\text{enzyme}\;\text{method}\right)\right)}^{-1.094}\\ \times {\left(\text{age}\right)}^{-0.287} \end{array}$$

The other method was the one reported by Horio as the following equation for men:

$$\begin{array}{l} \mathrm{GFR}\;\left(\text{Horio}\right)\;\left(\mathrm{mL}/\min\right)=\left(33-0.065\times \mathrm{age}-0.493\times \mathrm{BMI}\right)\\ \;\times \text{Weight}/\text{Serum}\;\text{creatinine}\;\left(\text{enzyme}\;\text{method}\right)\\ \;\times 14.4 \end{array}$$

Trained interviewers obtained information on smoking status, drinking status, medical history, use of antihypertensive agents, and use of medication for diabetes mellitus. HbA_1c_ (as defined by NGSP, the National Glycohemoglobin Standardization Program) was calculated with the following equation, which was recently proposed by a working group of the Japanese Diabetes Society (JDS):

$${\mathrm{HbA}}_{1c}\left(\mathrm{NGSP}\right)\;=\;{\mathrm{HbA}}_{1c}\left(\mathrm{JDS}\right)\;+\;0.4\%$$

[8]

Presence of diabetes was defined as HbA_1c_ (NGSP) ≥ 6.5%, and/or initiation of glucose-lowering medication or insulin therapy [9].

Measurement of CIMT by ultrasonography of the left and right common carotid arteries was performed by two medical doctors (NT and MN) using a LOGIQ Book XP with a 10-MHz transducer (GE Healthcare, Milwaukee, WI, USA) that was programmed with IMT measurement software, Intimascope (Cross Media Ltd., Tokyo, Japan) [10]. The values of right and left CIMT without measurement of plaque were calculated and the maximum CIMT value was used for analysis. Since a previous study reported the normal CIMT value as < 1.1 mm we defined atherosclerosis as CIMT ≥ 1.1 mm [11].

### Statistical analysis

Differences in age-adjusted mean values or prevalence of potential confounding factors by height quartile were calculated and logistic regression models were used for calculating odds ratios (OR) and 95% confidence intervals (CI) for the association of carotid atherosclerosis with height. In addition, subjects were stratified by BMI status because higher BMI is associated with greater CIMT [4,12]. Since the World Health Organization (WHO) has agreed on BMI ≥ 25 kg/m^2^ as an international classification for being overweight [13], we adopted 25 kg/m^2^ as the BMI cutoff point.

Two different approaches were used for making adjustments for confounding factors. First, the data were only adjusted for age. Second, we included other possible confounding factors, namely smoking status (never smoker, former smoker, current smoker), alcohol consumption (non-drinker, current light to moderate drinker (one to six times/week), current heavy drinker (every day)), body mass index (kg/m^2^), diabetes mellitus (no/yes), systolic blood pressure (mmHg), antihypertensive medication use (no/yes), serum triglycerides (mg/dL), serum HDL-cholesterol (mg/dL), serum AST (IU/L), and serum creatinine (mg/dL).

All statistical analyses were performed with the SAS system for Windows (version 9.3; SAS Inc., Cary, NC, USA). All *P*-values for statistical tests were two-tailed.

## Results

The clinical characteristics of the study population are summarized in Table 1. The mean age was 64.8 years (±10.7 SD). Table 1 shows age-adjusted characteristics for this study population according to height levels. Current drinker status, serum creatinine level and GFR estimated with the Horio method (mL/min) were significantly positively associated with height, while GFR estimated with the JCKD method (mL/min/1.73 m^2^) was inversely associated with height. Even the age-adjusted mean values of JCKD-estimated GFR and height showed a significant inverse association, but further adjustments for height and weight reversed the association. The adjusted mean values were 65.0 ml/min/1.73 m^2^ for the first quartile (Q1; < 158.7 cm), 66.4 mL/min/1.73 m^2^ for the second quartile (Q2; 158.7 to 163.0 cm), 69.5 mL/min/1.73 m^2^ for the third quartile (Q3; (163.1 to 167.9 cm), and 74.8 mL/min/1.73 m^2^ for the fourth quartile (Q4; > 167.9 cm) (*P* for trend = 0.011). A comparison of its associations with various body heights showed that estimated GFR was not an effective tool for evaluating kidney function.

**Table 1:** Age-adjusted mean values of patient characteristics according to height

	**Quartiles of height**				***P***
	**Q1 (low)**	**Q2**	**Q3**	**Q4 (high)**	
Men					
Median height, cm	155.6	161.0	165.1	170.1	
Number at risk	331	349	321	336	
Age, years	69.6 ± 9.5	67.3 ± 8.9	63.2 ± 10.5	59.1 ± 10.5	
Systolic blood pressure, mmHg	142	143	141	143	0.629
Antihypertensive medication use, %	27.9	27.3	25.7	27.4	0.921
Body mass index, kg/m^2^	23.6	23.8	23.6	23.6	0.772
Current drinker, %	42	52	55	54	0.004
Current smoker, %	28	25	26	24	0.704
Serum triglycerides (TG), mg/dL	121	124	129	127	0.613
Serum HDL-cholesterol (HDL), mg/dL	55	54	55	54	0.795
Serum aspartate aminotransferase (AST), IU/L	25	26	25	24	0.099
Diabetes, %	8.0	9.3	10.7	9.3	0.721
Serum creatinine, mg/dL	0.83	0.89	0.91	0.92	< 0.001
Estimated GFR (JCKD; mL/min/1.73 m^2^)	74.4	69.0	67.3	65.3	< 0.001
Estimated GFR (Horio; mL/min)	81.4	81.8	84.1	88.1	< 0.001

Age: mean ± standard deviation; *P*: *P* factor; height quartiles: < 158.7 cm (Q1), 158.7 to 163.0 cm (Q2), 163.1 to 167.9 cm (Q3), and > 167.9 cm (Q4); GFR: glomerular filtration rate.

Of the total of 1,337 men, 312 were diagnosed with carotid atherosclerosis.

Table 2 shows the ORs and 95% CIs for carotid atherosclerosis (CIMT ≥ 1.1 mm) according to height for all subjects, demonstrating that there was no significant association between these two factors.

**Table 2:** Odd ratios (OR) and 95% confidence intervals (CI) for risk of atherosclerosis in relation to height for all subjects

	**Height quartiles**					**For one SD increment in height**
	**Q1 (low)**	**Q2**	**Q3**	**Q4 (high)**	***P *** **for trend**	
Carotid atherosclerosis (CIMT ≥ 1.1 mm)						
All subjects						
Number at risk	331	349	321	336		
Number of cases (percentage)	101 (31)	95 (27)	68 (21)	48 (14)		
Age-adjusted OR	1.00	1.02 (0.72 to 1.45)	0.94 (0.64 to 1.37)	0.77 (0.51 to 1.17)	0.229	0.99 (0.86 to 1.15)
Multivariable OR	1.00	0.92 (0.64 to 1.32)	0.83 (0.56 to 1.23)	0.65 (0.42 to 1.01)	0.053	0.92 (0.79 to1.07)

Multivariable OR: adjusted further for age, systolic blood pressure, antihypertensive medication use, body mass index, smoking, alcohol intake, diabetes, serum triglycerides, serum HDL-cholesterol, serum aspartate aminotransferase (AST) and serum creatinine; carotid atherosclerosis defined as CIMT ≥ 1.1 mm; height quartiles: < 158.7 cm (Q1), 158.7 to 163.0 cm (Q2), 163.1 to 167.9 cm (Q3), and > 167.9 cm (Q4).

Table 3 shows the ORs and 95% CIs for carotid atherosclerosis (CIMT ≥ 1.1 mm) according to height stratified by BMI status. We detected a significant inverse association between height and carotid atherosclerosis for overweight participants but not for non-overweight participants.

**Table 3:** Odd ratios (OR) and 95% confidence intervals (CI) for atherosclerosis in relation to height stratified by body mass index (BMI)

	**Height quartiles**					**For one SD increment in height**
	**Q1 (low)**	**Q2**	**Q3**	**Q4 (high)**	***P *** **for trend**	
Carotid atherosclerosis (CIMT ≥ 1.1 mm)						
Overweight (BMI ≥ 25 kg/m^2^)						
Number at risk	93	121	102	115		
Number of cases (percentages)	36 (39)	38 (31)	23 (23)	15( 13)		
Age-adjusted OR	1.00	0.75 (0.42 to 1.35)	0.61 (0.32 to 1.18)	0.43 (0.21 to 0.88)	0.018	0.75 (0.58 to 0.97)
Multivariable OR	1.00	0.69 (0.37 to 1.28)	0.54 (0.27 to 1.08)	0.37 (0.17 to 0.79)	0.008	0.71 (0.54 to 0.94)
Non-overweight (BMI < 25 kg/m^2^)						
Number at risk	238	228	219	221		
Number of cases (percentages)	65 (27)	57 (25)	45 (21)	33 (15)		
Age-adjusted OR	1.00	1.14 (0.74 to 1.77)	1.15 (0.72 to 1.84)	1.03 (0.61 to 1.72)	0.845	1.13 (0.94 to 1.35)
Multivariable OR	1.00	1.06 (0.67 to 1.68)	1.09 (0.67 to 1.78)	0.87(0.51 to 1.49)	0.724	1.05 (0.87 to 1.27)

Multivariable OR: adjusted further for age, systolic blood pressure, antihypertensive medication use, body mass index, smoking, alcohol intake, diabetes, serum triglycerides, serum HDL-cholesterol, serum aspartate aminotransferase (AST) and serum creatinine; carotid atherosclerosis defined as CIMT ≥ 1.1 mm; height quartiles: < 158.7 cm (Q1), 158.7 to 163.0 cm (Q2), 163.1 to 167.9 cm (Q3), and > 167.9 cm (Q4).

We also investigated the effects on atherosclerosis of interaction between height and two BMI categories, BMI ≥ 25 kg/m^2^ and BMI < 25 kg/m^2^, and observed significant interaction between height and BMI category, and the multivariable-adjusted *P* for the effect of this interaction on atherosclerosis was 0.035.

## Discussion

A major finding of the present study of Japanese men was that, even though no significant associations between height and carotid atherosclerosis assessed in terms of CIMT were observed among all subjects and non-overweight participants, an inverse association was observed for overweight participants.

Previous prospective studies showed height was inversely associated with incidence of or mortality from cardiovascular disease [1,2,5].

The Japan Public Health Center (JPHC)-based prospective study of 15,564 men and women aged 40 to 59 showed an inverse association of height with incidence of stroke; the multivariable hazard ratio (95% CI) for stroke for an increment in height of one SD (6.43 cm for men, 5.79 cm for women) was 0.82 (0.74 to 0.90) [1].

Another study with 1,289 Japanese men aged 60 to 74 years reported that an increase in CIMT was an independent risk factor for stroke: the multivariable-adjusted relative risk (95% CI) for the highest compared with the lowest quartiles of maximum CIMT was 3.0 (1.1 to 8.3) [3].

On the other hand, a study with 96 healthy men aged 18 to 45 years reported that the parameter most strongly correlated with CIMT is BMI (r = 0.338, *P* < 0.001) [12].

In our study presented here, we observed no significant association between height and carotid atherosclerosis for all subjects. However, we found evidence that height was inversely associated with carotid atherosclerosis but only for overweight men.

The reason why this association was restricted to overweight men warrants discussion. One study indicated that CIMT correlated significantly with endothelial dysfunction [14], which is believed to be one of the most important initial steps in the atherosclerosis process [15], and some components of the metabolic syndrome have been reported to be associated with endothelial dysfunction [16]. Therefore, compared to the non-overweight participants in our study, the overweight participants may already have been at higher risk of progression of atherosclerosis. The physical effect of short stature may offer an explanation for the independent association between height and CIMT. Short stature is associated with faster heart rates and shortened return times for reflected waves and augmentation of the primary systolic pulses, thus leading to increased central aortic pressure [17], while other studies have reported a positive relationship between systolic blood pressure and CIMT [18]. Short stature may thus be associated with increased CIMT. Birth weight and height show a strong correlation [19] and low birth weight is known to be associated with altered renal shape, reduced renal volume, and fewer nephrons [20]. Thus, short stature may increase the risk of renal malfunction and hypertension [21] which also may lead to higher risk of increased CIMT. However, in our study, associations between height and carotid atherosclerosis assessed in terms of CIMT, were observed even after adjustment for serum creatinine. Throughout the fetal period and childhood, environmental factors such as nutrition and socioeconomic circumstances deeply affect childhood development [22] and malnutrition in childhood can be associated with short stature and poor health. A study with two and a half-year-old children showed a higher protein intake was associated with lower blood pressure [23], and another study of children (> two years) reported blood pressure was higher in malnourished children and in those who had recovered from malnutrition after an average period of six years [24]. This indicates that malnutrition in childhood leads to atherosclerosis and arteriosclerosis via enhanced blood pressure.

Possible limitations of this study warrant consideration. Because we did not have access to creatinine clearance data and estimated GFR is not an effective tool for evaluating kidney function for a comparison of associations with various body heights [25], we could not perform an analysis adjusted for precise renal function. However, our study showed that associations between height and carotid atherosclerosis remained significant even after adjustment for serum creatinine. In addition, height is known to be subject to diurnal variations which might result in misclassification into height category. However, our survey was performed during daytime only, so that the risk of misclassification was probably minimized. Furthermore, we also analyzed the association between a one SD increment in height and carotid atherosclerosis as was done in previous studies [1,5,25] and found that the associations were significant. Since a previous study reported that the mean height for the age range 30 to 39 was lower for men born between 1936 and 1945 (163.8 cm) than for those born between 1961 to 1970 (170.6 cm) [2], this difference might be due to a change in diet and food consumption from during to after the Second World War. Since Q1 was the oldest age group, it was not unexpected that carotid atherosclerosis was most prevalent in Q1 and least prevalent in Q4, which was the youngest age group. However, the associations between height and carotid atherosclerosis were the same, even when we limited the analysis to older subjects who were born during or before 1945; the multivariable adjusted ORs of carotid atherosclerosis for an increment of one SD in height were 0.86 (0.72 to 1.02) for total subjects, 0.62 (0.45 to 0.86) for overweight (BMI ≥ 25 kg/m^2^) and 0.98 (0.80 to 1.21) for non-overweight (BMI < 25 kg/m^2^) subjects. We also observed significant interaction between height and BMI category for those subjects; the multivariable adjusted *P* for the effect of this interaction on carotid atherosclerosis was 0.033. However, we could not establish any causal relationships because this was a cross-sectional study.

## Conclusion

In conclusion, even though this study of Japanese men found no significant association between height and carotid atherosclerosis (CIMT ≥ 1.1 mm) for all and non-overweight participants (BMI < 25 kg/m^2^), an inverse association was established for overweight participants with (BMI ≥ 25 kg/m^2^).

## Abbreviations

AST: Serum aspartate aminotransferase; BMI: Body mass index; CI: Confidence interval; CIMT: Carotid intima-media thickness; HbA1c: Hemoglobin A1c; JCKDI: Japanese Chronic Kidney Disease Initiative; JDS: Japanese Diabetes Society; JPHC: Japan Public Health Center; NGSP: The National Glycohemoglobin Standardization Program; OR: Odds ratio; TG: Triglyceride; WHO: World Health Organization.

## Competing interests

The authors declare that they have no competing interests.

## Authors’ contribution

YS carried out the design of the study and performed the statistical analysis, interpreted the data, and drafted or revised the manuscript. MN, TS and KK designed the study, were involved in data collection, and checked the manuscript. HG, SS, HY, NT, KA, and KA participated in the study concept and checked the manuscript. TM was a general coordinator and designed the study. All authors read and approved the final manuscript.
